# Do trypanosome turncoats wait before they commit?

**DOI:** 10.7554/eLife.03176

**Published:** 2014-06-03

**Authors:** Cher-Pheng Ooi, Gloria Rudenko

**Affiliations:** 1 **Cher-Pheng Ooi** is in the Division of Cell and Molecular Biology, Imperial College London, London, United Kingdom; 2 **Gloria Rudenko** is in the Division of Cell and Molecular Biology, Imperial College London, London, United Kingdomgloria.rudenko@imperial.ac.uk

**Keywords:** *Trypanosoma brucei*, variant surface glycoprotein (VSG), parasites, antigenic variation, expression site attenuation, developmental reprogramming, other

## Abstract

The strategy that sleeping sickness parasites use to evade the mammalian immune system may be linked to the metamorphosis that allows them to transfer from mammals into tsetse flies.

**Related research article** Batram C, Jones NG, Janzen CJ, Markert SM, Engstler M. 2014. Expression site attenuation mechanistically links antigenic variation and development in *Trypanosoma brucei*. *eLife* **3**:e02324. doi: 10.7554/eLife.02324

**Image** The parasite that causes sleeping sickness can switch between different surface coats as it multiplies in the mammalian bloodstream

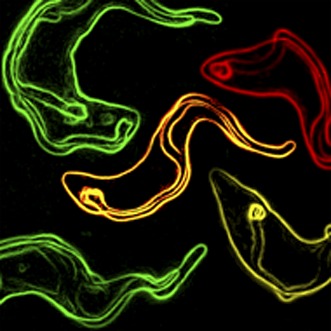

African trypanosomes cause sleeping sickness in humans, which is potentially lethal if left untreated. However, in order to spread to new hosts, these deadly parasites must be transmitted by tsetse flies, and this need to alternate between life in the mammal and life in the insect has made their biology unique. This includes the ability to undergo a metamorphosis from a form that rapidly proliferates within the bloodstream to a non-proliferative form that is prepared for life in the fly (Figure 1A; MacGregor et al., 2011, 2012). Normally, this change takes place when the concentration of the proliferative form of the parasite in the bloodstream reaches a certain density.

**Figure 1.**
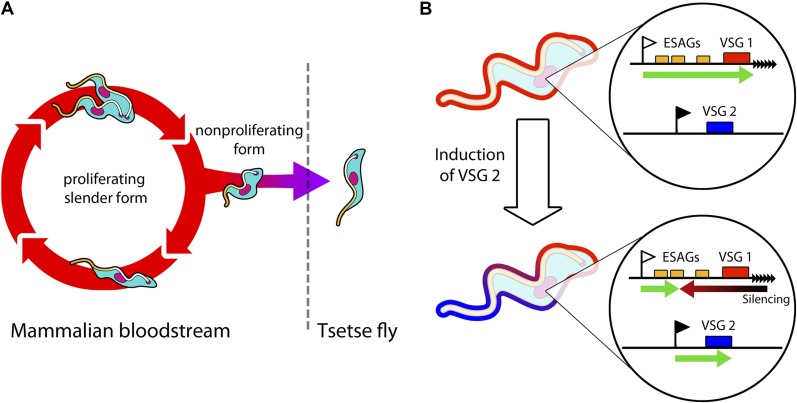
Trypanosomes must radically change in order to survive in both mammals and tsetse flies. (**A**) Trypanosomes proliferating in the mammalian bloodstream change into a non-proliferative form that is capable of transferring into a tsetse fly. All bloodstream trypanosomes have a dense surface VSG coat. (**B**) Trypanosomes express VSG proteins from a VSG expression site at a chromosome end (black triangles). The VSG expression sites include expression site associated genes (ESAGs; yellow boxes) as well as the telomeric VSG genes (red box). In the top panel, VSG1 is being expressed, with transcription (green arrow) proceeding from a promoter (white flag). Batram et al. found that inducing parasites to express a different VSG—labelled here as VSG 2 (blue box)—from another genomic location (black flag) results in a repressive gradient silencing the active expression site (red arrow). This not only silences VSG 1, but also reduces the expression of ESAGs near VSG 1.

In the mammal, trypanosomes are covered with a protective coat made of variant surface glycoprotein (VSG). In order to avoid detection by host antibodies, trypanosomes repeatedly switch to new VSG coats (Horn and McCulloch, 2010). Now, in *eLife*, Markus Engstler and colleagues from the University of Würzburg—including Christopher Batram as first author—argue that the process of switching the VSG coat could play a central role in both helping the trypanosome to avoid the mammalian immune system and helping it to escape from the mammal into the insect (Batram et al., 2014).

In the bloodstream, trypanosomes express only one VSG coat at a time out of a repertoire of hundreds. The active VSG is expressed in a strictly mutually exclusive fashion from an expression site located at the end of a chromosome (Figure 1B). Batram et al. forced the trypanosome to express a different VSG from elsewhere in the genome. Surprisingly, a repressive gradient that extends from the chromosome end resulted in the active VSG expression site shutting down. This silencing was dependent on a histone methyltransferase (Figueiredo et al., 2008), indicating that chromatin remodelling was involved. Amazingly, this expression site silencing also appeared to trigger trypanosomes to change from the proliferative form that undergoes continuous division in the bloodstream to become the non-proliferative form that is adapted for life in the insect.

The VSG expression sites also contain expression site associated genes (ESAGs; Hertz-Fowler et al., 2008). Using genetic techniques to knock down these genes also triggered the trypanosome to change from the proliferative to the non-proliferative form. It therefore appears that some expression site associated genes could function as ‘sensors’ that cue trypanosomes to become quiescent when the expression site shuts down. This suggests that VSG expression site silencing—which could potentially be reversed—may be involved in trypanosomes preparing for life in the tsetse fly.

This reveals an interesting and unexpected connection between the silencing of the active VSG expression site and the escape of trypanosomes out of the infected host. As silencing the active expression site is reversible, the proliferating form of the parasite may be able to adopt a ‘wait and see’ state before committing to becoming non-proliferative. This challenges the paradigm that commitment to a non-proliferative form depends on the population density of the parasites, and is an irreversible process.

However, these experiments raise more questions than they answer. It is not clear if this causative link between expression site silencing and progression through the life cycle also occurs in a natural infection. Alternatively, it could be a byproduct of forcing the trypanosome to express high levels of a second surface coat gene. How often would a trypanosome normally find itself in a situation where high levels of a second VSG are expressed, but not the associated ESAGs?

Batram et al. provide illuminating insight into how trypanosomes can silence their active VSG expression site, and show that this can proceed directionally from the chromosome end as well as from the promoter (Figure 1B). However, Batram et al. also propose an unexpected link between VSG coat switching and how the parasite progresses through its life cycle. When trypanosomes reach high densities in the mammalian host, do they follow predetermined genetic programming in the hope that they will be picked up by a tsetse fly? Or alternatively, can trypanosomes reversibly probe various options? The answers will certainly prove informative as to how this seemingly simple single-celled parasite outsmarts its more highly evolved hosts.
